# Can Mouse Imaging Studies Bring Order to Autism Connectivity Chaos?

**DOI:** 10.3389/fnins.2016.00484

**Published:** 2016-11-10

**Authors:** Adam Liska, Alessandro Gozzi

**Affiliations:** ^1^Functional Neuroimaging Laboratory, Center for Neuroscience and Cognitive Systems @ UniTn, Istituto Italiano di TecnologiaRovereto, Italy; ^2^Center for Mind/Brain Sciences, University of TrentoRovereto, Italy

**Keywords:** fMRI, functional connectivity, autism, mouse, resting-state, DMN, CNTNAP2, pathoconnectomics

## Abstract

Functional Magnetic Resonance Imaging (fMRI) has consistently highlighted impaired or aberrant functional connectivity across brain regions of autism spectrum disorder (ASD) patients. However, the manifestation and neural substrates of these alterations are highly heterogeneous and often conflicting. Moreover, their neurobiological underpinnings and etiopathological significance remain largely unknown. A deeper understanding of the complex pathophysiological cascade leading to aberrant connectivity in ASD can greatly benefit from the use of model organisms where individual pathophysiological or phenotypic components of ASD can be recreated and investigated via approaches that are either off limits or confounded by clinical heterogeneity. Despite some obvious limitations in reliably modeling the full phenotypic spectrum of a complex developmental disorder like ASD, mouse models have played a central role in advancing our basic mechanistic and molecular understanding of this syndrome. Recent progress in mouse brain connectivity mapping via resting-state fMRI (rsfMRI) offers the opportunity to generate and test mechanistic hypotheses about the elusive origin and significance of connectional aberrations observed in autism. Here we discuss recent progress toward this goal, and illustrate initial examples of how the approach can be employed to establish causal links between ASD-related mutations, developmental processes, and brain connectional architecture. As the spectrum of genetic and pathophysiological components of ASD modeled in the mouse is rapidly expanding, the use of rsfMRI can advance our mechanistic understanding of the origin and significance of the connectional alterations associated with autism, and their heterogeneous expression across patient cohorts.

## The connectivity theory of autism: open questions and controversies

Autism is a heterogeneous syndrome characterized by core behavioral features including deficits in social communication and interaction, as well as restricted, repetitive patterns of behavior, and interests (Association AP, 2013). Although a primary and unitary etiology for autism spectrum disorder (ASD) has not been identified, its high heritability has been consistently documented, revealing a contribution of complex and highly heterogeneous genetic mutations (Geschwind, 2009; Geschwind and State, 2015; Sanders et al., 2015). Remarkably, although previously identified mutations, genetic syndromes, and *de novo* copy number variations (CNVs) account for about 10–20% of ASD cases, none of these single known genetic causes accounts for more than 1–2% of cases (reviewed in Abrahams and Geschwind, 2008). The phenotypic expression (i.e., “penetrance”) of these genetic components is also highly variable, ranging from fully penetrant point mutations to polygenic forms with multiple gene–gene and gene–environment interactions. Remarkable variability exists also in the extent of cognitive and behavioral abnormalities presented by affected individuals (Georgiades et al., 2013; Lai et al., 2014; Chang et al., 2015), making heterogeneity a dominant theme for this group of disorders.

The advent of non-invasive brain imaging raised hopes that such clinical heterogeneity could be narrowed down to a small number of identifiable “imaging endophenotypes” that could help ASD diagnosis, patient stratification, and possibly provide clues as to the elusive etiology of this group of disorders. Unfortunately, the results of imaging studies have proven overall as variable as the clinical manifestations of ASD (Stanfield et al., 2008; Ecker et al., 2015). A notable exception to this scenario was the initial observation of reduced connectivity between brain regions in ASD patients, a finding first reported by Horwitz et al. (1988) using PET, and later corroborated by task-based (Just et al., 2004) and resting-state fMRI (rsfMRI) studies (Cherkassky et al., 2006; Kennedy and Courchesne, 2008; Assaf et al., 2010), which revealed impaired long-range synchronization in spontaneous brain activity. Together with evidence of reduced white matter connectivity detected with MRI (reviewed in Anagnostou and Taylor, 2011), these observations form the basis of the so called “under-connectivity theory of autism” (Anagnostou and Taylor, 2011; Just et al., 2012), according to which deficient long-range communication between brain regions may underlie ASD symptoms and pathophysiology. However, recent imaging studies have strongly challenged this view, highlighting a much more heterogeneous picture (see Vasa et al., 2016 for a recent review). For example, rsfMRI mapping in a large cohort of patients has revealed the presence of concomitant hypo- and hyper-connectivity (Di Martino et al., 2014), although a clear prevalence of hypo-connected regions was apparent. Similarly, widespread hyper-connectivity during childhood has also been recently described (Keown et al., 2013; Supekar et al., 2013; Uddin et al., 2013a), suggesting a possible neurodevelopmental origin for these alterations. More recently, the hypothesis that such conflicting findings could reflect greater inter-subject variability in ASD patients than in neurotypical controls (i.e., idiosyncratic connectivity) has been proposed (Hahamy et al., 2015). A putative confounding contribution of ASD-related motion and its effect on functional connectivity readouts is also the subject of an open controversy in the imaging community (Deen and Pelphrey, 2012; Power et al., 2012, 2015; Pardoe et al., 2016).

Collectively, the extensive literature published to date points at the presence of major functional connectivity alterations in ASD populations, although the identified regional patterns vary considerably across studies and patient cohorts (Kana et al., 2011; Müller, 2014; Ecker and Murphy, 2014; Ameis and Catani, 2015; Ecker et al., 2015; Bernhardt et al., 2016; Vasa et al., 2016). Despite this rapidly accumulating evidence, many fundamental questions as to the origin and significance of connectional alterations in ASD remain unanswered. For one, the neurophysiological underpinnings of these connectional aberrancies are largely unknown, and a causal etiopathological contribution of specific genetic variants to impaired connectivity in ASD remains to be firmly established. More broadly, it is unclear whether these abnormalities are a causative or epiphenomenal consequence of the disease, and whether their heterogeneous expression reflects cohort effects, different genetic etiologies, or neurodevelopmental trajectories. The exact relationship between connectivity alterations and the severity of ASD manifestation remains also obscure, with the vast majority of the human neuroimaging literature being focused on high-functioning ASD cohorts (Vissers et al., 2012).

A deeper understanding of the origin and significance of these phenomena is greatly complicated by our very limited understanding of the neurobiological foundations of macro-scale neuroimaging readouts commonly employed in ASD research, such as white matter microstructural parameters (e.g., fractional anisotropy, Owen et al., 2014) or the elusive functional couplings underlying rsfMRI-based functional connectivity. This has left us with a major explanatory gap between mechanistic models of brain function at the cellular and microcircuit level, and the emergence of macroscale functional activity in health and pathological states such as those that are observed in autism. As a result, we are currently unable to properly interpret and back-translate clinical evidence of aberrant connectivity into interpretable neurophysiological events/models that can help understand, diagnose or treat these disorders. It is also becoming apparent that a full disambiguation of the multifactorial and complex determinants of aberrant functional connectivity in ASD can only be obtained through the combined use of refined clinical imaging methods and multimodal-multiscale investigational approaches that currently can only be applied in experimental animal models.

## Bridging the gap: functional connectivity mapping in mouse autism models

The identification of several high-confidence ASD-risk genes involved in syndromic forms of autism (Sanders et al., 2015) has been paralleled by the generation of mouse lines recapitulating human mutations. Despite predictable limitations in reliably modeling the full phenotypic spectrum of a complex (and possibly only human) developmental disorder like ASD, mouse models can be harnessed to understand how genetic alterations translate into relevant changes in cells and circuits, and ultimately to identify points of convergence for molecular pathways, cells, circuits, and systems that may result in a deeper understanding of the pathophysiology of ASD and related behavioral deficits (Arguello and Gogos, 2012; Nelson and Valakh, 2015; Vasa et al., 2016). For example, molecular investigations in ASD mouse models have been instrumental in the identification of a limited set of molecular pathways to which ASD-involved genes seem to converge, including, among others, synaptogenesis, synaptic function, and neuronal translational regulation (reviewed in de la Torre-Ubieta et al., 2016). This effort has been accompanied by the development of ASD-relevant behavioral phenotyping assays, primarily targeted at social, communication, and repetitive behaviors (Silverman et al., 2010a; Wöhr and Scattoni, 2013; Kas et al., 2014; Homberg et al., 2016). Interestingly, many—but not all—models showed autism-like traits, with manifestations ranging from repetitive behaviors to reduced social communication (ultrasonic vocalizations) and social interest (reviewed in Ellegood and Crawley, 2015). However, despite the widespread application and high face validity of ASD behavioral phenotyping, the significance and translational relevance of mouse behavioral alterations to human ASD remain debated (Wöhr and Scattoni, 2013) and should be extrapolated with caution.

Recent advances in mouse rsfMRI mapping (reviewed in Gozzi and Schwarz, 2016) offer the opportunity of extending mouse modeling of ASD to the investigation of the neurobiological underpinnings and etiopathological significance of ASD-related connectivity aberrations. Specifically, improvements in MRI imaging hardware, together with tighter control of physiological and motion artifacts (Weber et al., 2006; Ferrari et al., 2012) have led to robust and reproducible identification of homotopic rsfMRI networks covering known cortical and subcortical systems in the mouse by several research groups (Mechling et al., 2014; Nasrallah et al., 2014; Sforazzini et al., 2014; Zerbi et al., 2015; Shah et al., 2016). Interestingly, distributed networks encompassing heteromodal prefrontal and posterior cortical regions have also been identified (Sforazzini et al., 2014; Zerbi et al., 2015; Shah et al., 2016), leading to the suggestive hypothesis of the presence of evolutionary precursors of the human salience network and default mode network (DMN) in this species (reviewed in Gozzi and Schwarz, 2016). This notion is empirically corroborated by the recent observation that cytoarchitecturally homologous regions such as anterior cingulate and retrosplenial cortices (Vogt and Paxinos, 2014) similarly serve as connectivity hubs in humans and mice (Cole et al., 2010; Tomasi and Volkow, 2011; Liska et al., 2015). Moreover, the application of rsfMRI to the mouse brain comes with several important advantages, including the possibility to use quantitative imaging modalities for an objective endo-phenotypic characterization of ASD-related pathology complementary to behavioral assays, and to validate its readouts with invasive techniques that are off limits for human research, including local field potentials (LFPs) coherence mappings (Zhan et al., 2014), local injection of neuronal tracers (Sforazzini et al., 2016), as well as an ever-increasing array of histopathological, stereological, or immunohistochemical post-mortem analyses.

**Figure 1:**
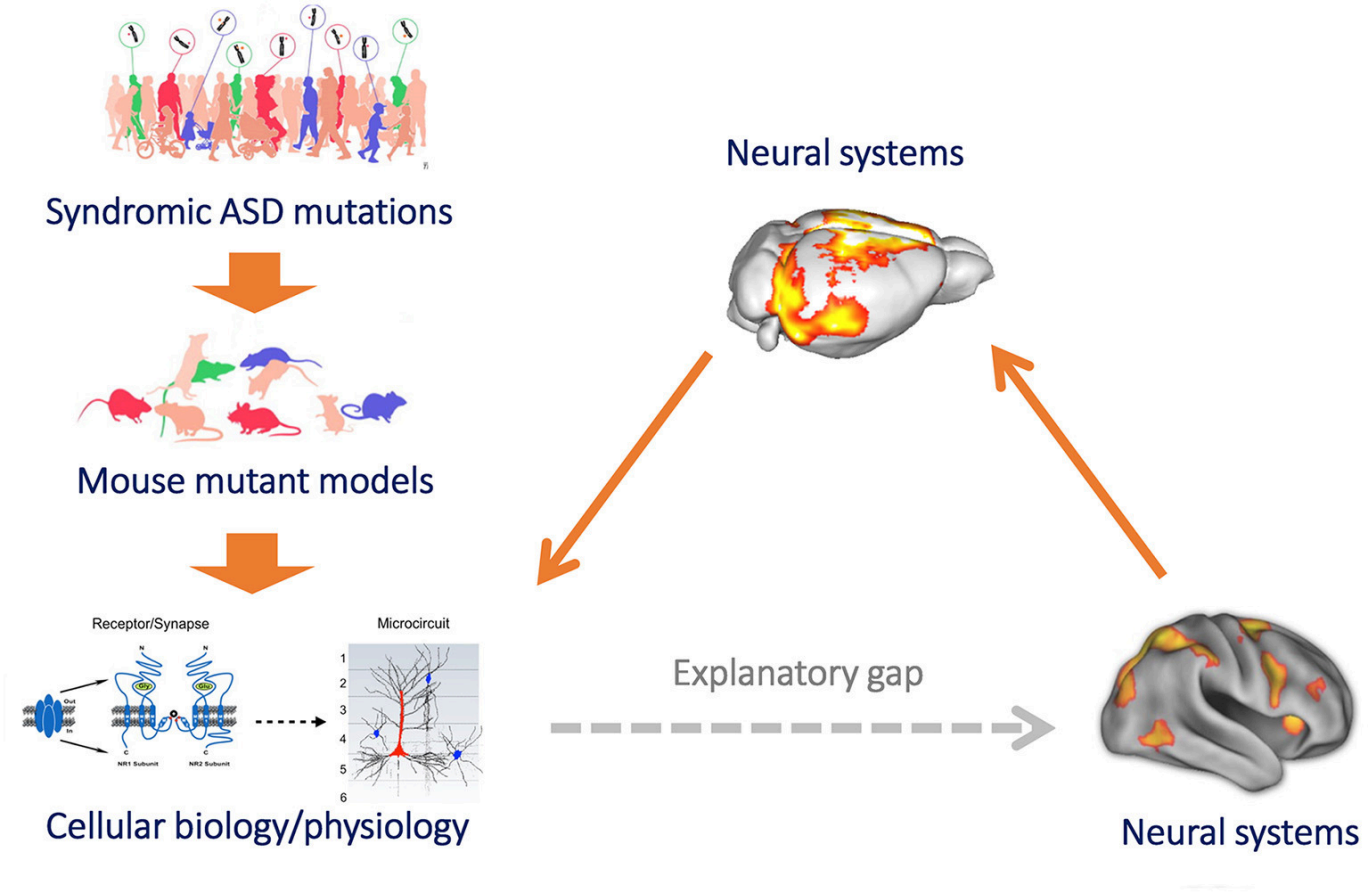
**Mouse imaging can bridge the gap between microscale models of brain function and clinical research of macroscale functional connectivity**. Mouse models provide a powerful reductive platform that can be employed to link etiological determinants of ASD, such as syndromic mutation or neurodevelopmental traits, to basic molecular and cellular signatures of pathology (left, top to down). However, until recently we have been unable to use this approach to study the neurobiological underpinnings of macroscale functional connectivity, owing to difficulty in translating models of brain function across levels of inquiry. This results in a major explanatory gap between clinical research (heavily relying on macroscale neuroimaging measures of brain function, such as rsfMRI) and preclinical neurobiological investigation in rodent models (bottom, right). The implementation of functional connectivity mapping via rsfMRI in the mouse (right) can bridge this gap, by permitting to causally relate connectional changes with basic molecular or cellular processes, and by permitting a direct translation of these findings from and to humans owing to the shared biophysical principle underlying these measurements (figure adapted from Arguello and Gogos, 2012; Anticevic et al., 2013 with permission).

Collectively, these correspondences strongly support the use of rsfMRI as a means to bridge research of functional connectivity aberrancies in autism across species (human vs. mouse) and levels of inquiry (from cellular- and microscale to meso- and macroscale, Figure 1), along two main investigational routes. First, rsfMRI can be used to establish *causal* (rather than *associative*) etiopathological contributions between specific ASD-associated genetic variants and macroscale connectivity, thus complementing analogous clinical research efforts using imaging genetics (Scott-Van Zeeland et al., 2010; Rudie et al., 2012). One notable experimental advantage of mouse imaging with respect to current human imaging genetic approaches is the possibility of mapping and comparing the effect of multiple mutations (via the use of different autism mouse models) under rigorously controlled experimental conditions, thus reducing the confounding contribution of experimental variables that can be only minimally controlled in human research, such as genetic and environmental variability, age (Uddin et al., 2013b), ASD-related motion, and group differences in cognitive states (Vasa et al., 2016). The main goal of this line of investigation is to assess whether seemingly unrelated ASD-risk mutations do converge on a limited number of distinct functional connectivity endophenotypes. An elegant demonstration of this approach has been recently described using morpho-anatomical MRI. Brain-volumetric phenotypes of 26 ASD mouse models as defined by structural MRI methods exhibited clustering into three main groups, each with a distinct set of concomitant changes in size across different brain regions (Ellegood et al., 2015). Such reduction of morpho-anatomical heterogeneity is not surprising, given the wide (and sometimes opposing) stream of pathophysiological alterations observed in syndromic forms of autism, which range from basic molecular or synaptic mechanism such as protein synthesis (Geschwind and Levitt, 2007; Auerbach et al., 2011) up to homeostatic regulations of excitatory and inhibitory neurotransmission (Nelson and Valakh, 2015). Analogous analyses with regards to functional connectivity phenotypes should be possible in the future to associate basic pathophysiological traits with macroscale connectional aberrancies.

A second main line of investigation is the combined use of mouse rsfMRI and multiscale neurobiological techniques to obtain a mechanistic description of ASD-related phenotypes and pathophysiological pathways leading to aberrant functional connectivity. This research can include, but is not limited to, a deeper investigation of syndromic ASD mutations associated with specific pathological traits [e.g., Tuberous Sclerosis 2 as a key mediator of impaired autophagy and increased synaptic density (Tang et al., 2014)], and can possibly be extended to investigate risk factors that have been also more loosely implicated in autism. This research effort may generate crucial mechanistic information that can be used to back-translate clinical evidence of aberrant connectivity into interpretable neurophysiological events/models that can help understand, diagnose, or treat these disorders. A brief description of initial steps toward these two main goals is reported in the next two sections.

## Functional connectivity mapping in genetic models of autism

An outstanding question in ASD connectivity studies is whether genetic mutations associated with syndromic forms of autism are sufficient to produce aberrant macroscale functional connectivity. Initial mouse rsfMRI studies seem to corroborate this hypothesis. Specifically, Haberl and colleagues have recently investigated functional and structural connectivity in the Fmr1^−/y^ model of fragile X syndrome (FXS; Budimirovic and Kaufmann, 2011) and described connectional aberrations in sensory networks (Haberl et al., 2015). These included reduced structural integrity of the corpus callosum and an increase in local connectivity of the primary visual cortex, as probed by viral tracers, an effect accompanied by reduced rsfMRI coupling between visual and other neighboring sensory cortical regions. The authors suggested that the observed decoupling could explain sensory processing defects that are often observed in FXS patients (Boyd et al., 2010).

In another recent study, homozygous mice lacking the ASD-risk gene CNTNAP2 (Peñagarikano et al., 2011) exhibited reduced long-range and local functional connectivity in cingulate and prefrontal regions (Liska et al., 2016), two key heteromodal areas of the mouse brain previously characterized as functional connectivity hubs, owing to their rich connectivity with other brain areas (Liska et al., 2015). Interestingly, impaired antero-posterior prefrontal connectivity between components of the mouse DMN was associated with reduced social investigation, a behavioral measure regarded as a core “autism trait” in mice (Wöhr and Scattoni, 2013). This finding recapitulates analogous imaging results obtained in human carriers of CNTNAP2 gene polymorphisms (Scott-Van Zeeland et al., 2010), hence providing a first example of the translational value of this approach. This finding is consistent with the presence of impaired GABAergic neurotransmission in these animals (Peñagarikano et al., 2011), a trait that could result in aberrant oscillatory rhythms. It is interesting to note that analogous prefrontal hypo-connectivity has been observed using rsfMRI in BTBR mice, an idiopathic model of autism characterized by agenesis of the corpus callosum and by analogous excitatory/inhibitory imbalances (Sforazzini et al., 2016).

rsfMRI mapping has also been recently carried out in a mouse model of human 15q13.3 microdeletion, a CNV associated with schizophrenia, intellectual disability, and ASD (Shinawi et al., 2009). Compared to wild-type mice, 15q13.3 mice showed widespread patterns of hyper-connectivity along the hippocampal-prefrontal axis, a network commonly affected in schizophrenic patients (Gass et al., 2016). Notably, Gass and colleagues also showed that aberrant functional connectivity could be acutely rescued by pharmacological stimulation of nicotinic acetylcholine alpha 7 receptors, in keeping with a contribution of this mechanism to the development of schizophrenia-related phenotypes in these mice (Gass et al., 2016). Although the phenotypic traits of this mouse line appear to be more closely related to schizophrenia rather than to ASD (Fejgin et al., 2014), the results of this study are important as they show that CNVs and genetic alterations with partial penetrance to ASD could produce divergent connectional phenotypes (e.g., hyper- and hypo-connectivity), suggesting a plausible contribution of genetic heterogeneity to some of the discrepant imaging findings in humans. Importantly, these initial mouse studies argue against an artifactual (e.g., motion-driven) origin of connectivity aberrations reported in human ASD research, because the use of light sedation in mice along with artificial ventilation allows for the acquisition of virtually motion-free images.

## Neurobiological pathways leading to aberrant functional connectivity

A few recent studies have provided important mechanistic investigations of ASD-relevant phenotypes associated with aberrant functional connectivity. In the first of such studies, Zhan et al. (2014) investigated whether deficits in synaptic pruning, a putative pathophysiological determinant of autism (Hutsler and Zhang, 2010), result in connectivity alterations. To probe this hypothesis, the authors measured rsfMRI connectivity in Cx3cr1^KO^ mice, a mouse line characterized by microglia-dependent synaptic pruning deficits as a result of deficient neuronal-microglia signaling (Paolicelli et al., 2011). Synaptic pruning deficits in Cx3cr1^KO^ were found to be associated with long-range functional connectivity impairments, a finding corroborated by LFPs coherence recordings in freely-behaving animals. Interestingly, the authors also showed that impaired pruning was associated with core mouse “autism traits,” and that long-range fronto-hippocampal connectivity was a good predictor of social behavior. This study is of special importance, as it was the first to suggest a role for dysfunctional synaptic maturation in shaping long-range functional synchronization and to postulate a contribution of immune system mediators to this cascade. Empirical evidence in support of this hypothesis comes from another recent study (Kim et al., 2016), where analogous phenotypes where observed in mice characterized by defective autophagy in microglia, including increased synaptic density, impaired social activity, and a trend for impaired connectivity between posterior-sensory and prefrontal regions. Similarly, Filiano et al. (2016) recently showed that deficiency in interferon-γ, a key immune signaling protein, is associated with social deficits and frontal rsfMRI hyper-connectivity in SCID mice, thus corroborating a putative mechanistic link between immune dysfunction, impaired social behavior, and functional connectivity. Although promising and mechanistically relevant, these initial results should be extrapolated to autism research with great caution, as a pathophysiological contribution of immune and microglia deficits to ASD has yet to be unambiguously demonstrated (Estes and McAllister, 2015). They, however, powerfully illustrate how the combined use of rsfMRI, mouse genetics and state-of-the-art neuro-biological approaches can elucidate pathways leading to aberrant functional connectivity, an approach that can be extended to investigate the role of multiple ASD-relevant pathophysiological factors, including syndromic genetic mutations.

## Limitations and future perspectives

Like any other experimental approach, mouse rsfMRI is accompanied by limitations that should be taken into account when the approach is used to investigate the basis of connectivity alterations in ASD. First and foremost, as mouse rsfMRI experiments normally employ sedation to minimize stress and motion of animals during scans, the contribution of possible genotype-dependent differences in sensitivity to anesthesia (Petrinovic et al., 2016) should be controlled. The fact that to date only a minority of studies (Zhan et al., 2014; Liska et al., 2016; Sforazzini et al., 2016) have reported genotype-dependent measures of anesthesia sensitivity is a factor for concern, as differences in anesthesia depth/sensitivity can affect connectivity strength and distribution of the imaged networks (Nasrallah et al., 2014). The impact of anesthesia *per-se* as a putative modifier of intrinsic connectional architecture appears to be less of an issue, as a large body of human and rodent research shows that, under light controlled sedation, the regional patterns of functional correlation seem to be largely preserved (reviewed in Gozzi and Schwarz, 2016). As pointed out in previous work, a rigorous control of motion and physiological state is also of paramount importance to obtain reliable network mapping (Jonckers et al., 2015; Gozzi and Schwarz, 2016). It should also be mentioned that, although the field is still lacking in standardized protocols and methods that would facilitate comparison of experimental results across studies and sites, this issue is receiving increased attention and collaborative efforts are underway to address it.

The initial studies described here represent only the first step toward a greater understanding of the origin and underpinnings of connectional alterations in ASD. Future investigations are required to describe commonalities and differences between brain functional networks in the mouse and human from multiple points of view, including topology (Sporns and Betzel, 2016; van den Heuvel et al., 2016a), biological underpinnings (Richiardi et al., 2015; Wang et al., 2015; van den Heuvel et al., 2016b), and functional equivalence (Li et al., 2015). Similarly, studies of additional genetic etiologies associated with ASDs, covering heterogeneous pathophysiological pathways, are crucial to achieve a deeper understanding of whether the connectional signatures are mutation specific or can be regarded as a generalizable phenomenon. When coupled to analogous clinical efforts aimed at identification of connectional aberrancies in genetically homogeneous populations [e.g., 16p11.2 deletion (Simons Vip Consortium, 2012; Owen et al., 2014)], the method can also be used to investigate the cellular and physiological basis of clinically relevant neuroimaging readouts and, via a comparison between human and mouse imaging findings, to obtain an assessment of the translational and construct validity of mouse models of ASD. The developmental trajectory of these alterations could in principle also be investigated in mouse models, although critical limitations in the accuracy of physiological control in young mice and pups exist.

Much of mouse ASD modeling has been so far primarily addressed at monogenic ASD syndromes, which represent ~10% of ASDs (Silverman et al., 2010a; Nelson and Valakh, 2015). The recapitulation, in mice, of high-confidence genetic etiologies associated with ASD offers the opportunity to probe specific hypotheses about circuit dysfunction and ASD pathology that can be directly extrapolated to homologous clinical populations [e.g., 16p11.2 microdeletion (Simons Vip Consortium, 2012; Owen et al., 2014)]. An important limitation of current ASD translational research is its inability to reliably model “idiopathic” autism, which is the most frequent diagnostic label for ASD-related behavioral manifestations. Attempts to use forward genetic approaches in inbred mouse lines exhibiting ASD-like behaviors without a specific genetic determinant have been proposed, with the inbred BTBR mouse line probably being the most notable example in the field (Silverman et al., 2010b; Gogolla et al., 2014; Squillace et al., 2014). Translational relevance of neuro-behavioral findings obtained by comparing genetically homogeneous inbred lines like asocial BTBR and “normosocial” B6 mice is, however, debated (Dodero et al., 2013; Squillace et al., 2014). Nevertheless, novel neuromolecular approaches and the use of induced pluripotent stem cells (iPSCs) from patients have begun to reveal common downstream neurobiological pathways in idiopathic forms of autism characterized by shared neuroanatomical features [e.g., macrocephaly (Nicolini et al., 2015; Marchetto et al., 2016)]. Controlled manipulation of such signaling and molecular pathways in animal models is a foreseeable strategy that can be employed to expand our translational framework to the investigation of macroscale brain network aberrancies in idiopathic forms of ASD.

Finally, studies in which connectivity alterations are pharmacologically or genetically rescued may help clarify the relevance of functional connectional alterations to ASD pathology and its behavioral manifestations. Specifically, if connectivity alterations are an underlying cause of observed behavioral deficits, then behavioral phenotypic “rescue” should be accompanied by normalized patterns of brain functional connectivity. This research could indicate whether connectivity alterations are *necessary* for the expression of ASD-related behaviors in mice, or are instead an epiphenomenal manifestation of underlying pathophysiology, thus providing an empirical assessment of the pathophysiological relevance of connectivity aberrancies in ASD. “Rescue” studies may also help identify putative endo-phenotypes (complementary to behavior) that could serve as measurable readouts for early clinical translation and evaluation of novel ASD treatments in genetically defined autism syndromes (Smucny et al., 2014).

In conclusion, functional imaging of the mouse has now reached a turning point such that accurate modeling and investigation of ASD-connectivity aberrations is currently possible, via the use of readouts amenable to direct translation to human research (e.g., rsfMRI). Despite caveats, in the next few years the approach is poised to offer breakthroughs in our understanding of the pathogenesis of ASD-related connectivity aberrancies, possibly bringing some order to the intricate and often contradictory body of research detailing connectional alterations in patient populations.

## Author contributions

AG conceived and wrote the manuscript with input from AL.

### Conflict of interest statement

The authors declare that the research was conducted in the absence of any commercial or financial relationships that could be construed as a potential conflict of interest.
